# Deep learning for necrosis detection using canine perivascular wall tumour whole slide images

**DOI:** 10.1038/s41598-022-13928-1

**Published:** 2022-06-23

**Authors:** Taranpreet Rai, Ambra Morisi, Barbara Bacci, Nicholas J. Bacon, Michael J. Dark, Tawfik Aboellail, Spencer Angus Thomas, Miroslaw Bober, Roberto La Ragione, Kevin Wells

**Affiliations:** 1 Centre for Vision, Speech and Signal Processing, University of Surrey, Guildford, GU2 7XH UK; 2 School of Veterinary Medicine, University of Surrey, Guildford, GU2 7AL UK; 3 School of Biosciences and Medicine, University of Surrey, Guildford, GU2 7XH UK; 4 National Physical Laboratory, London, TW11 0LW UK; 5 Department of Veterinary Medical Sciences, University of Bologna, 40126 Bologna, Italy; 6 Fitzpatrick Referrals Oncology and Soft Tissue, Guildford, UK; 7 Department of Comparative, Diagnostic, and Population Medicine, College of Veterinary Medicine, University of Florida, Gainesville, FL USA; 8 Department of Microbiology, Immunology and Pathology, Colorado State University, Fort Collins, CO USA; 9 Department of Computer Science, University of Surrey, Guildford, GU2 7XH UK

**Keywords:** Image processing, Machine learning, Sarcoma

## Abstract

Necrosis seen in histopathology Whole Slide Images is a major criterion that contributes towards scoring tumour grade which then determines treatment options. However conventional manual assessment suffers from inter-operator reproducibility impacting grading precision. To address this, automatic necrosis detection using AI may be used to assess necrosis for final scoring that contributes towards the final clinical grade. Using deep learning AI, we describe a novel approach for automating necrosis detection in Whole Slide Images, tested on a canine Soft Tissue Sarcoma (cSTS) data set consisting of canine Perivascular Wall Tumours (cPWTs). A patch-based deep learning approach was developed where different variations of training a DenseNet-161 Convolutional Neural Network architecture were investigated as well as a stacking ensemble. An optimised DenseNet-161 with post-processing produced a hold-out test F1-score of 0.708 demonstrating state-of-the-art performance. This represents a novel first-time automated necrosis detection method in the cSTS domain as well specifically in detecting necrosis in cPWTs demonstrating a significant step forward in reproducible and reliable necrosis assessment for improving the precision of tumour grading.

## Introduction

Canine Soft Tissue Sarcoma (cSTS) are a heterogeneous group of mesenchymal neoplasms that derive from tissues of mesenchymal origin^1–6^. The anatomical site of cSTS varies significantly, but mostly involve the cutaneous and subcutaneous tissues^7^. Canine Soft Tissue Sarcoma (cSTS) are a large group that can be broken down into several subtypes, but are grouped together nonetheless, due to the similarities of microscopic and clinical features for each subtype. The general treatment of choice for cSTS is to surgically remove cutaneous and subcutaneous sarcomas, where they have a low re-occurrence rate after surgical excision. However, it is higher-grade tumours that can prove to be problematic leading to poorer prognosis and outcomes. Histological grade is the most important prognostic factor in human Soft Tissue Sarcoma (STS), and is likely one of the most validated criteria to predict outcome following surgery in canine patients^8–11^. It is widely accepted that the histological grading system for cSTS is applied to all cSTS subtypes to adopt simplicity. However, there can also be an inconsistent naming of subtypes which can lead to a poor correlation between classification of tumours and their histogenesis (tissue of origin). This sometimes results in confusion for pathologists, therefore, highlighting a need for standardisation^7^. Due to poor agreement when identifying sarcoma subtypes, we are focusing on one common subtype found in canines: canine Perivascular Wall Tumours (cPWT). Canine Perivascular Wall Tumours (cPWT) arise from vascular mural cells and can be recognisable from their vascular growth patterns which include staghorn, placentoid, perivascular whorling, and bundles from tunica media^12,13^.

The scoring for cSTS grading is broken down into three major criteria: differentiation, mitotic index and necrosis^7^. For the purposes of this paper, the study is focused on necrosis detection which is an important indicator of disease progression and its severity.

A sub-field of machine learning known as deep learning is used for necrosis detection in this work. Such deep learning algorithms are abundant in the medical imaging field and especially digital pathology, assisting in computer-aided diagnosis to classify images or automatically detect diseases. Deep learning has become increasingly ubiquitous and has proven to be very successful in recent image classification tasks in digital pathology^14–19^. The digitisation of histological slides into Whole Slide Images (WSI) has created the field of digital pathology. The field of digital pathology has allowed cellular pathology labs to move into digital workflows^20^. This has resulted in a change in working practices as clinical pathologists are no longer required to be present at the same location as pathology equipment. Potential benefits of this innovation includes remote working across borders, collaborative reporting, and the curation of large teaching databases. Nevertheless, several pathology tasks remain exposed to inter-observer variability, where two or more pathologists will differ in their assessment of a histological slide^16^. As a result, there is much interest in improving and automating pathology workflows whilst promoting standardisation for scoring certain criteria within grading, with greater reproducibility. Automatic necrosis detection in cSTS could decrease viewing times for the pathologists and reduce inter- and intra-observer variability, positively impacting accuracy in the tumour’s diagnosis and prognosis.

The study presented here aimed to classify regions demonstrating necrosis against regions that do not, in canine Perivascular Wall Tumour (cPWT) Whole Slide Images (WSIs), by using deep learning models such as pretrained Convolutional Neural Networks (DenseNet-161). In the literature, relatively few authors have investigated necrosis detection using machine learning methods. As necrosis detection is typically an image classification task, depending on the image resolution and ”field of view” (size of image), necrosis detection can be considered a texture detection problem. Earlier work in necrosis detection applied machine learning methods where texture features were used for Support-Vector Machine (SVM) classification^21^. The same authors later published literature where deep learning was compared with traditional computer vision machine learning methods in digital pathology. For necrosis detection, their proposed deep learning Convolutional Neural Network (CNN) architecture performed best with an average test accuracy of 81.44%^22^. Another set of authors investigated necrosis detection comparing both an SVM machine learning model and deep learning for viable and necrotic tumour assessment in human osteosarcoma WSIs^23^. The aim was to label the regions of WSI into viable tumor, necrotic tumor, and non-tumor. For evaluation the Volume Under the Surface score (VUS) was computed for non-tumour versus viable tumour versus necrotic. Their models produced 0.922 and 0.959 VUS scores for SVM and deep learning models respectively. Nevertheless, these works do not investigate canine Soft Tissue Sarcoma (cSTS) and so this paper addresses whether such deep learning models can also positively impact necrosis detection in cSTS.

Several methods of training deep learning models were investigated in this work, such as a pretrained DenseNet-161 (with and without augmentations), an extension of training this model via hard negative mining, to reduce false positive (FP) predictions and a stacking ensemble model. To the best of our knowledge this is the first work in automated detection of necrosis in cPWTs, as well as in cSTS and thus this methodology could be used for the necrosis scoring in an automated detection and grading system for cSTSs. To our best of knowledge, these results represent the highest F1-scores in regard to cPWT necrosis detection to date.

## Methods

### Data description and patch extraction process

A set of canine Soft Tissue Sarcoma (cSTS) histology slides obtained from the Department of Microbiology, Immunology and Pathology, Colorado State University were diagnosed by a veterinary pathologist. A senior pathologist at the University of Surrey confirmed the grade of each case (patient) and chose a representative histological slide for each patient. These slides were then digitised using a Hamamatsu NDP slide scanner (Hamamatsu Nanozoomer 2.0 HT) and viewed with the NDP.viewer platform. These slides were scanned at 40x magnification (0.23 m/pixel) with a scanning speed of approximately 150 s at 40x mode (15 mm $\times$ 15 mm) to create a digital Whole Slide Image (WSI).

**Figure 1:**
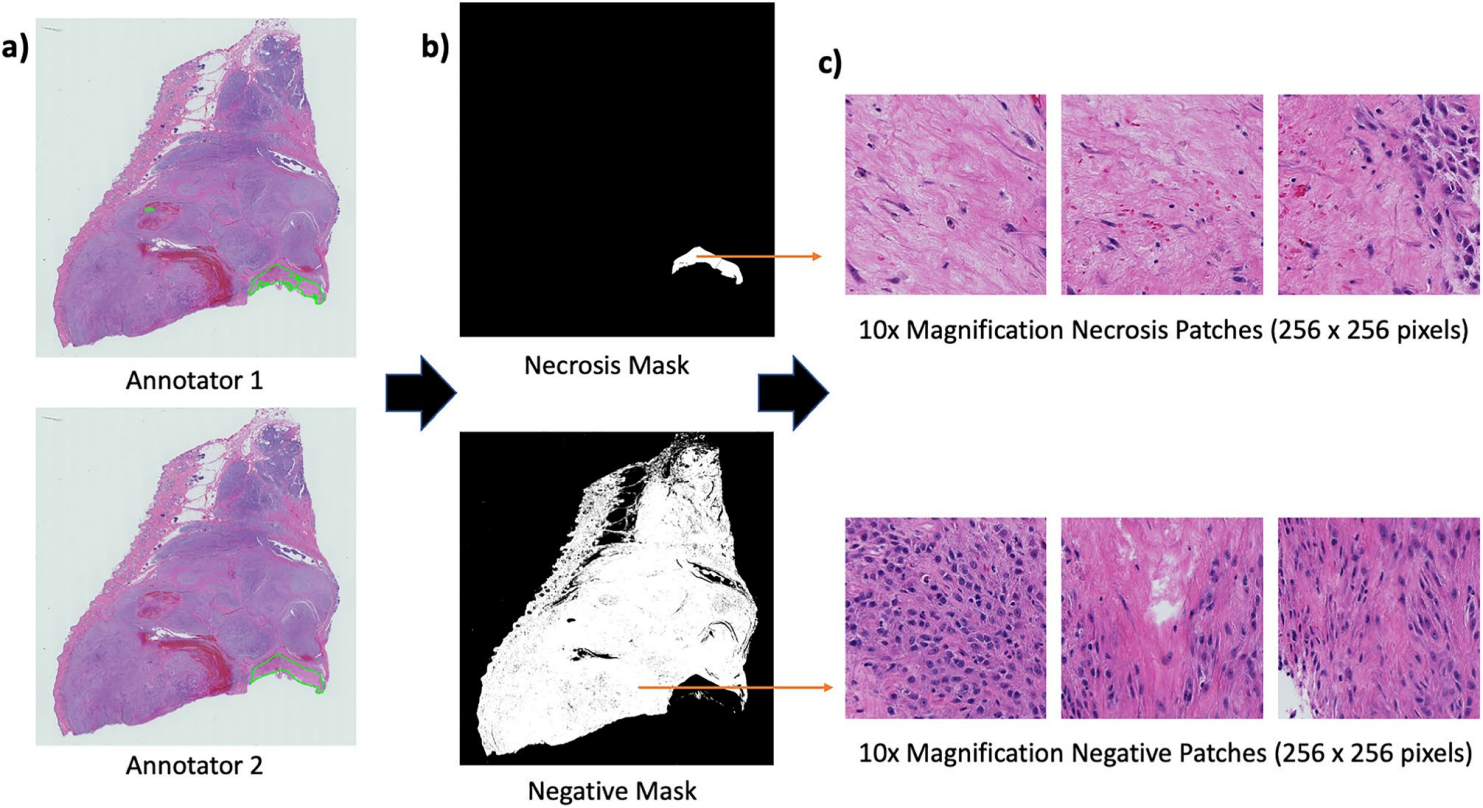
In (**a**), Annotations by ”Annotator 1” and ”Annotator 2” applied to the same canine Perivascular Wall Tumour (cPWT) Whole Slide Image (WSI). For the patch extraction process, binary masks (or maps) are generated, (shown in (**b**). A necrosis mask is created, highlighting the intersection agreement between both annotators, when considering a region as necrotic. Any disagreement is dismissed from the necrosis and negative binary masks. From applying Otsu thresholding we dismissed any non-tissue related regions and by using the intersection agreement for both annotators, we created a ”negative mask”, highlighting in white regions that do not contain necrosis. We used these masks to extract patches, as shown in (**c**). In this case we extract 10x magnification necrosis and negative patches of 256 $\times$ 256 pixels.

Two pathologists independently annotated the WSIs for necrosis using the open-source Automated Slide Analysis Platform (ASAP) software, as contours around the necrotic regions^24^. The pathologists used different magnifications (ranging from 5x to 40x) to analyse the necrotic regions before drawing contours. As a result, two class labels were created from these annotations: positive (necrosis) and negative, for subsequent analysis as a binary patch-based classification problem. In order to categorise a region as containing necrosis, both pathologist annotators needed to form an ”agreement”. Therefore, the intersection of the necrosis annotations were labelled as necrosis. Similarly, areas that are agreed to have no necrosis are labelled as negative. We used these annotations to create image masks for the patch extraction process and applied Otsu thresholding to remove non-tissue background from both classes creating tissue masks. A patch-based approach was applied due to the large nature of Whole Slide Images, which typically produce gigapixel images in the higher resolution layers of the pyramid format. Such large images cannot be directly fed into machine learning models and so patches of a smaller size are extracted from WSIs for further analysis. Using the aforementioned intersection of the annotator’s necrosis binary maps, non-overlapping patches of size 256 x 256 pixels were extracted from both necrosis and negative regions (2 classes). A demonstration of the patch extraction process can be visualised in Fig. 1.

The study investigated 10x magnification resolutions for necrosis detection as suggested by the on-board pathologists. The pathologists chose 10x magnification over 5x, 20x and 40x as the ideal resolution for necrosis detection. Non-overlapping patches of 256 by 256 pixels were extracted from regions of both classes using a minimum decision threshold for percentage of necrosis present in a patch. For a magnification of 10x, 30% of the patch must have contained necrosis pixels (determined from the expert defined labels) in order for it to be labelled as necrosis. A threshold of 30% for necrosis pixels was chosen as it was needed to take into account boundary effects of the necrosis clusters in the images. Patches extracted from boundaries of the necrosis cluster would almost certainly contain non-necrotic tissue. However, a suitable amount (in this case 30%) of necrosis tissue is required to sufficiently label a patch as necrosis for the effective training of deep learning models. A higher number would risk dismissing useful necrotic patches, whereas a lower number (thus more negative tissue in a patch) would likely cause confusion during the training of deep learning models.

### Deep learning model and experimental set-up

In order to evaluate the robustness and the veracity of our approach, we performed 3-fold cross validation, where a hold-out test set created to compare the models trained on the three different folds. In total we extracted patches from 32 patients (WSIs) to create our train, validation and test sets.

There were a total of 5784 necrosis patches from 20 slides for training/validation and 1151 necrosis patches from 12 slides for testing. Additionally, there were a total of 50,975 negative patches for training/validation and 31,351 negative patches for testing.

Class imbalance is apparent throughout the different folds of the datasets. To address the large variation of the negative class with a relatively small presence of the necrosis class, we reduced class imbalance by randomly extracting 800 negative patches per WSI and used these with all necrosis patches per WSI. This reduces the class imbalance to 1:4 for necrosis to negative, respectively, although not excessively. Weighted cross entropy loss was applied to mitigate class imbalance. It was found that the models trained with this level of class imbalance performed marginally better. Therefore, we opt to train with this mild imbalance ratio, as balancing the dataset to 50/50 may risk throwing away useful (vital) information from the negative class.

**Figure 2:**
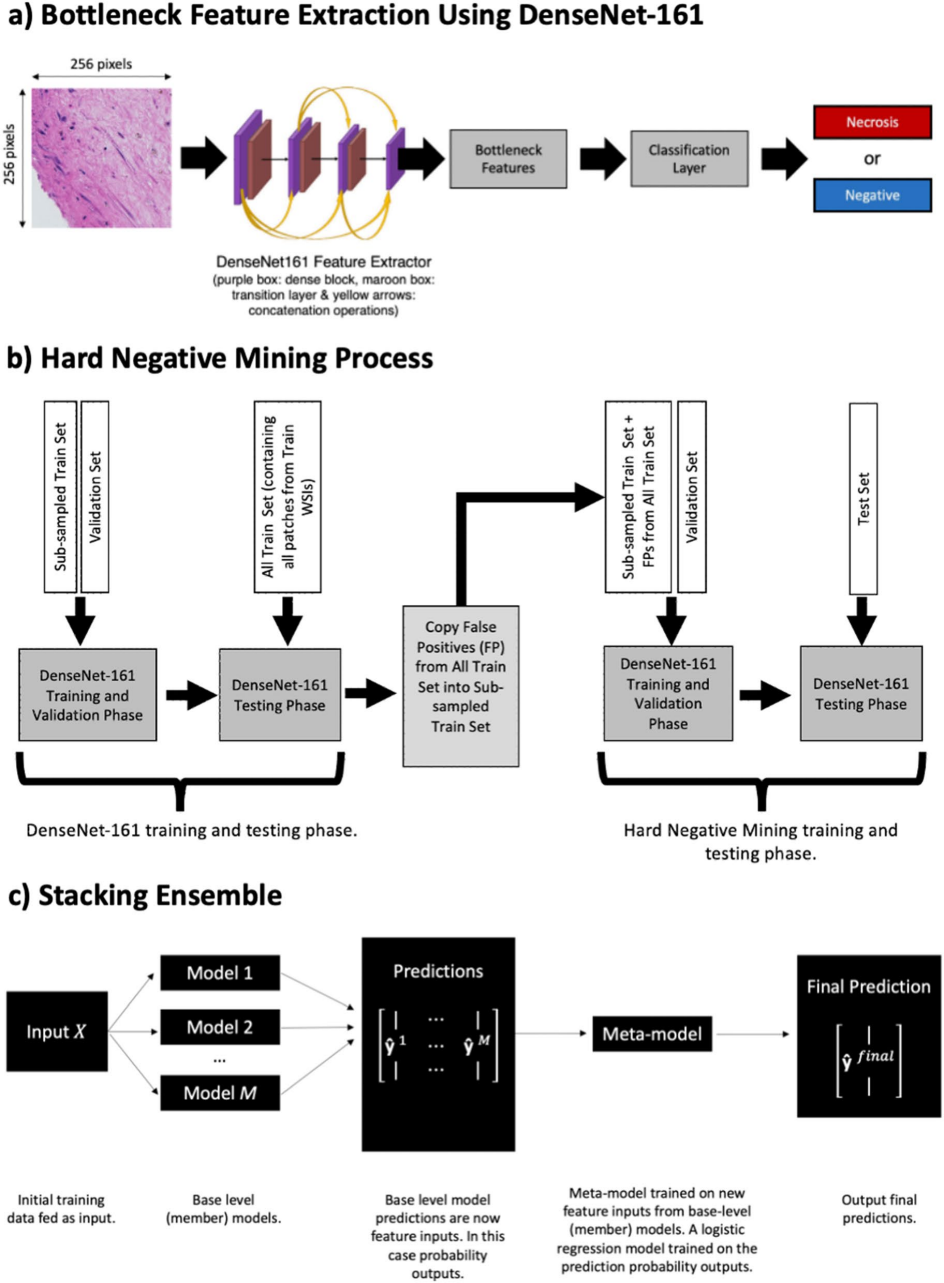
(**a**) Bottleneck feature extraction using DenseNet-161. A patch size of 256 x 256 pixels is fed into a DenseNet-161 feature extractor, where bottleneck features are obtained. These features are then fed into a classification layer for further training and validation, classifying necrosis or negative patches. (**b**) Hard negative mining approach to train the model with additional ”difficult” examples presented to the network. (**c**) Stacking ensemble. The input *X* is fed into *M* base-level member models: DenseNet-161 model, the DenseNet-161 model with augmentations and the hard negative mining model. The prediction outputs of these models $\hat{y}_M$ are combined and fed into a logistic regression meta-model as new feature inputs. New coefficients are learnt in this logistic regression model, before final predictions are output $\hat{y}^{final}$.

The deep learning model implemented transfer learning bottleneck feature extraction. Previous investigations of several pretrained networks including VGG and ResNet architectures have been shown to have a positive impact in digital pathology^25,26^. However, DenseNet-161 was chosen due to its leading performance in previous works^27^. According to one study, DenseNet-161 can be used for fast and accurate classification of digital pathology images to assist pathologists in daily clinical tasks^28^. Thus, bottleneck features were extracted from DenseNet-161, producing an output of 2208 features. These features were then fed into a classification layer, to classify ”necrosis” or the ”negative” class per patch. As DenseNet has been pretrained on ImageNet, its standard output is for 1000 classes. However, as necrosis detection is considered a binary problem, a binary classification layer has been used that replaces the original multiclass classification layer. See part a Fig. 2.

Comparative experiments were implemented comparing the pretrained DenseNet-161 to Sharma et al’s proposed CNN model and an AlexNet^22^. At the initial 50% decision threshold, these models produce higher f1-scores. However, it should be noted that the sensitivities of the AlexNet and proposed CNN models were lower than our previously implemented DenseNet-161 models. Our DenseNet-161 model also provided a higher AUC value prior to thresholding and post-processing (See Supplementary Table S1). Therefore, the pretrained DenseNet-161 was the primary baseline choice of model for this paper, as it had a greater capacity for further performance improvement via thresholding and post-processing.

A grid search was previously implemented in preliminary experiments to determine optimal hyperparameters. The range of hyperparameters investigated were batch sizes of 16, 32 and 48, learning rates of 0.00001, 0.0001, 0.001 and 0.01 and different variations of scheduler steps. As a result, for all experiments a batch size of 32 was inputted into each model and the loss function used was cross entropy loss. The Adam optimiser initialised with a learning rate of 0.0001 was used, with a scheduler step of 20 and a scheduler gamma of 0.5 as optimal values^29^. This optimised set of hyperparameters resulted in a smooth, stable training behaviour. We calculated the RGB mean and standard deviation values per fold for patch image normalisation. For every fold, each model was trained for 100 epochs, where the model from the epoch with the lowest validation loss was automatically chosen as the best performing model. This selected model was then applied to both the validation and test sets for evaluation during training and final testing, respectively.

The models were implemented in Python, using the PyTorch deep learning framework. Other notable Python packages used for pre-processing and post-processing tasks included OpenSlide, NumPy, Pandas, OpenCV and Math. Both training and testing of the deep learning models were performed using GPU programming. The hardware and resources available for implementation used a Dell T630 system, which included 2 Intel Xeon E5 v4 series 8-Core CPUs at 3.2 GHz, 128 GB of RAM, 4 nVidia Titan X (Pascal, Compute 6.1, Single Precision) GPUs.

### Other types of models investigated

In this section we describe the different type of deep learning and ensemble models investigated for necrosis detection. Apart from the ensembles, all deep learning models used the same hyperparameters and training scheme as described in the previous section.

#### DenseNet-161 with augmentations

Adding augmented patch images to a training set is a common strategy to mitigate the lack of variation and size of limited datasets^30^. Modifications were thus applied to an existing image to produce new images, using random horizontal/ vertical flips and colour jitter (random changes to the brightness, contrast, saturation and hue of a patch image). A change of upto/minus 40% is randomly applied for brightness, contrast and saturation.

#### Hard negative mining model

Hard negative mining was performed in order to reduce the number of false positives. This is an approach that allowed us to train the model with additional ”difficult” examples presented to the network^31^. Firstly, a ”full” training set was created, where we extracted every single negative class patch from the training set. Secondly, we then applied the best model on the full training set to infer a new set of predictions. The model with the lowest validation loss was selected as the best model. Any prediction on a patch that was a false positive (and did not exist in the original dataset) was added to the sub-sampled dataset; thus creating a new dataset known as the ”hard negative training set”. Lastly, we then trained the DenseNet-161 model using this new dataset and evaluated as before. A flow diagram of the implementation of the hard negative experiments can be visualised in part b of Fig. 2.

#### Ensemble model

A common approach to boost the performance of machine learning models is via the employment of ensemble models where the ensemble process is depicted in part c of Fig. 2. The basic concept of ensembles is to train multiple models (or base-member models) and combine their predictions into one single output^32^. Ensemble models typically outperform single models (individual base-member models) on their respective target datasets. This can be seen in recent machine learning based competitions such as on the Kaggle platform and MICCAI^33–35^.

The ensemble model was trained on the sub-sampled training dataset. To make use of all the data from the training WSIs, we made inferences on the full training set patches (including the original training set patches). There are various methods and combinations to create ensemble models, however, preliminary experiments demonstrated that an ideal combination consisted of base-member models was the DenseNet-161 model, the DenseNet-161 model with augmentations and the hard negative mining model.

Preliminary experiments investigated combining ensemble predictions and training a logistic regression model (as a meta-model) on the full training set of patches to produce prediction probability outputs. We then tested this trained logistic regression model on validation and hold-out test sets. Pseudocode for the stacking ensemble is also represented in Algorithm 1. 
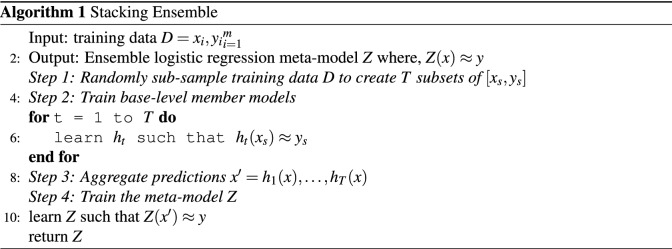


### Post-processing

It is important to note that although sensitivity is a vital measure in the medical domain, the number of false positives greatly influences the score for necrosis, thus impacting overall grading. For our problem, both the precision and sensitivity were considered to be equally as important and therefore the F1-score was used to determine the optimal thresholds for each folds validation. These thresholds were then applied to our hold-out test set for each fold. Additionally, for comparison, the mean optimal threshold for the three folds was computed and applied this to our hold-out test set. The F1-score is the harmonic mean between the precision and sensitivity. It takes into account both the sensitivity and precision producing a weighted average of the two metrics. Both precision (formula 2) and sensitivity (formula 3) contribute equally to the F1 score (formula 5):

1 $$\begin{aligned}&Accuracy = \frac{TP + TN}{TP + TN + FN + FP} \end{aligned}$$

2 $$\begin{aligned}&Precision = \frac{TP}{{TP + FP}} \end{aligned}$$

3 $$\begin{aligned}&Sensitivity = \frac{TP}{{TP + FN}} \end{aligned}$$

4 $$\begin{aligned}&Specificity = \frac{TN}{{TN + FP}} \end{aligned}$$

5 $$\begin{aligned}&\mathrm {F1} = 2 * \frac{Sensitivity * Precision}{{Sensitivity + Precision}} \end{aligned}$$

where TP, FP and FN are true positives, false positives and false negatives, respectively.

**Figure 3:**
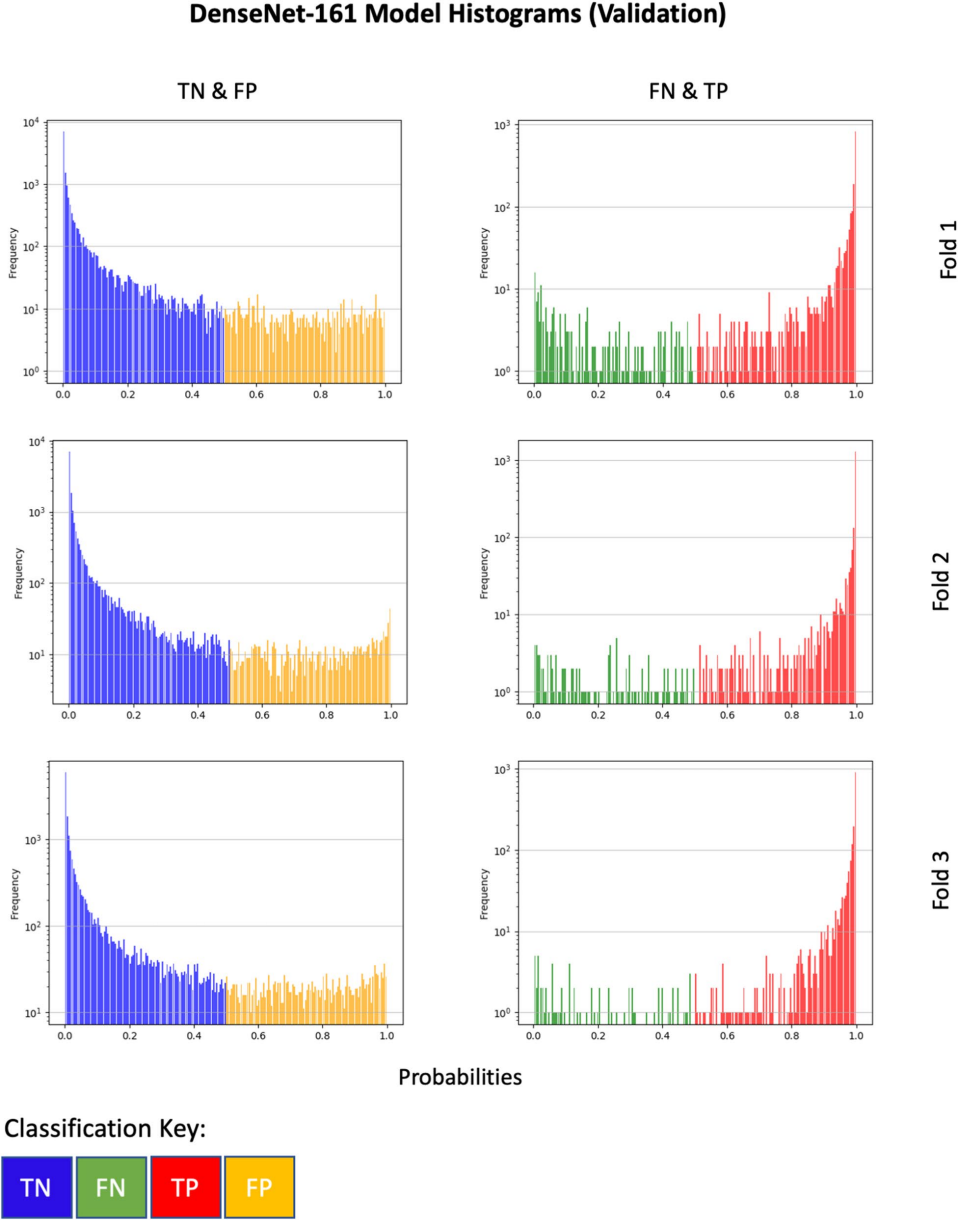
Histograms of the initial classification results based on a standard 0.5 (50%) probability decision threshold. Depicted are true negatives (TN), false positives (FP), false negatives (FN) and true positives (TP) for the DenseNet-161 model. On the left side depicts histogram plots of TN and FP for each validation fold, whereas on the right side depicts histogram plots of FN and TP. These combinations were chosen for the plots as they complement each other. It can be seen that all three folds are characteristically similar in distribution. TN and TP predictions typically produce high probabilities, as can be seen by the frequency of such predicted probabilities. Increasing the probability threshold would increase the number of true negatives and reduce the number of false positives. However, this would subsequently increase the number of false negatives and reduce the number of true positives.

Figure 3 depicts histograms showing true positives, true negatives, false positives and false negatives for the DenseNet-161 model. The y-axis is log-normalised to compress and better visualise the frequency of predictions as the number of true negatives significantly outweigh the number of true positives. The x-axis (probabilities) is split into 100 bins. The left side of this figure shows true negatives (TN) and false positive (FP) histograms for each validation fold, whereas on the right depicts histogram plots of false negatives (FN) and true positives (TP). The validation set was used to choose optimal thresholds. The hold-out test set was used as a data set purely for evaluation and not contribute towards any change in strategy (i.e. to prevent data/information leaks). These classification output combinations were chosen as they complement one other. For example, increasing the probability decision threshold would increase the number of TNs and reduce the number of FPs. However, this would subsequently increase the number of FNs thus reducing the number of TPs. For all folds, the TN and FP plots shows a wide range of prediction probabilities, with a heavy skew towards the lower probabilities. The FN and TP plot for fold 3 (validation) appears to shows slight sparsity among FN predictions in comparison to other folds.

**Figure 4:**
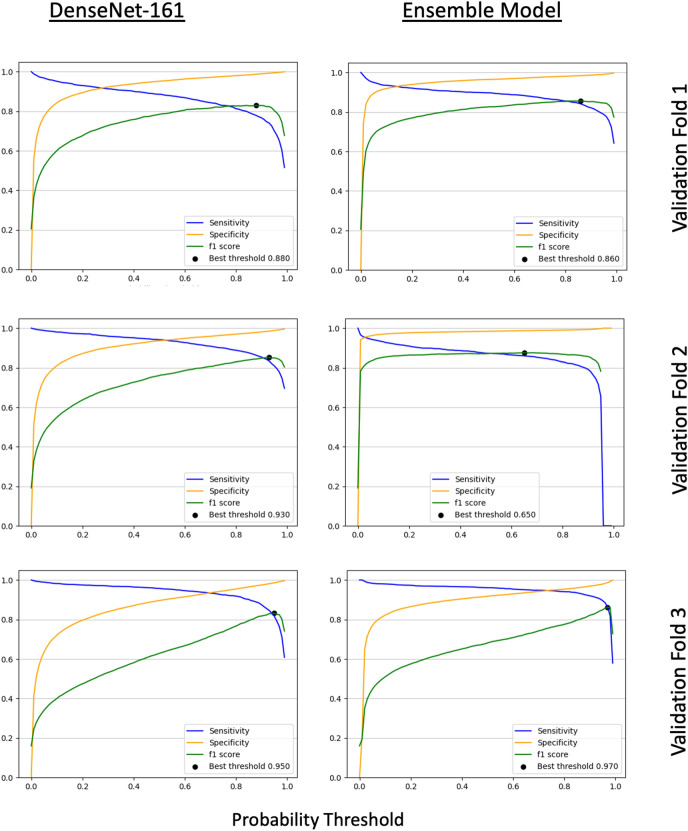
Line graphs that depict the sensitivity, specificity and weighted F1-score calculated for each probability threshold, for the three validation folds from the DenseNet-161 and ensemble models. To determine the optimal probability threshold, we choose the threshold with the highest F1-score. In the above plots, these are denoted as ”Best threshold”. For example, for the ensemble model, in validation fold 1, this threshold was 0.86, for fold 2 it was 0.65 and for fold 3 it was 0.97.

The sensitivity, specificity and F1-scores were calculated for several probability thresholds for each validation fold, as shown in Fig. 4. For both the DenseNet-161 model and the ensemble model, it was apparent that high probability thresholds had an adverse effect on sensitivity. The F1-score was used as the metric to determine optimal thresholds.

The probability thresholds *t* ranged from 0.01 to 1 and so choosing the optimal validation threshold *T* for the F1-score *F*1 can be represented formally as:

6 $$T = \mathop{\arg\,\max}\limits_t F1(t)$$

In general, the DenseNet-161 model followed a similar trend for all 3 folds where the optimal threshold was high (between 0.88 and 0.93). The ensemble model demonstrated similar results apart from fold 2 where the optimal probability decision threshold was found to be 0.65.

**Figure 5:**
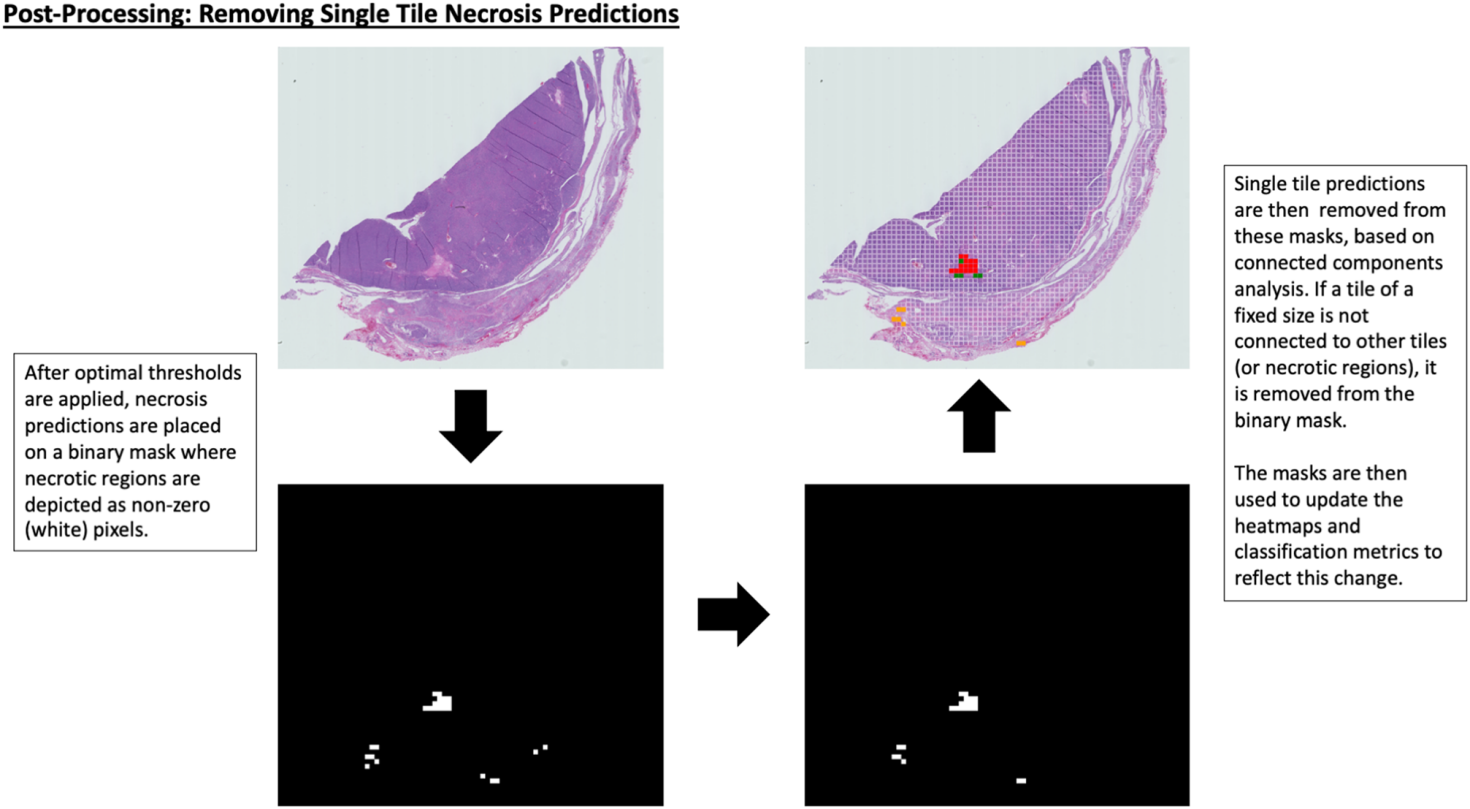
The post-processing step to remove predicted single necrosis tiles is depicted. The necrosis predictions are applied to a binary mask which is a downsized binary map of the original WSI, by a factor of 32x, for computational efficiency. Connected components analysis is subsequently performed, where if a tile of a fixed size (in this case an area of 32 pixels squared) is not connected to other tiles, horizontally, vertically, or diagonally, it is removed from the mask. Final predictions are updated based on using these binary masks.

Necrosis detection in WSIs often displays sporadic false positive predictions. From domain knowledge and discussions with the on-board pathologists, it was determined that single tile (patch) necrosis predictions in a WSI would typically not be considered necrotic in most circumstances, if surrounded by non necrotic tissue. This is due to the size of the isolated region playing a part in quantifying on whether a region should be considered necrosis and scored. Of course, single patches could be necrotic, however, this would be analogues to outlier detection or statistical noise and fluctuations. As a result, a post-processing step was applied to remove these single tile necrosis predictions. The necrosis predictions are applied to a binary mask, which is has dimensions downsized by 32x compared with the original WSI. Connected components analysis is then performed^36^. In this case, if a tile of a fixed size (32 $\times$ 32 pixels) is not connected to any other neighbouring tiles (or necrotic regions), horizontally, vertically, or diagonally, it is automatically removed from the mask. As a result, the final predictions are updated based using these binary masks. This process is depicted in Fig. 5.

## Results

### Individual base-member model results

Results for the DenseNet-161 model, DenseNet-161 with augmentations model and the hard negative mining model are presented in Table 1. It must be denoted that these results are patch-based and not WSI (or patient) based. Therefore, classification results are based on whether a sampled patch is considered necrotic or not. Furthermore, these results are based on a default ”decision threshold” or simply ”threshold” of 50%. This means that if a patch is predicted to have a probability confidence of more than 50%, then it is considered necrosis. Anything less than 50% is considered negative (not necrotic).

The addition of augmentations appeared to show a marginal improvement in sensitivity as shown with the validation (italicised) and hold-out test sets. The hard negative mining model demonstrated an improvement on the F1-score and specificity scores across the 3 folds, compared to the DenseNet-161 model for both the validation and test sets. However, sensitivity was adversely affected across the average of the validation and test sets.

Nevertheless, across all models, sensitivity scores were higher in the test set than validation. This is most likely due to the models finding unencountered tissue types to be suspicious during test, a consequence of training with limited datasets.

The highest validation and test specificity was produced by the ensemble model; the model trained on the combined probability outputs of the DenseNet-161 model, DenseNet-161 with augmentations model and the hard negative mining model. Consequently, the highest validation and test F1-scores are also from the ensemble model. As the F1-score is used as a basis for choosing the best performing model, we continued with the ensemble model and used the DenseNet-161 model as a comparison for further post-processing.

**Table 1:** 3-fold averaged results for the DenseNet-161 model, the hard negative mining model and the DenseNet-161 with augmentations model, with reported mean sensitivity, specificity and F1-scores averaged across all three folds.

Model	Set	Sensitivity		Specificity		F1 score	
DenseNet-161	Validation	0.928	$+$ 0.029	0.928	$+$ 0.024	0.724	$+$ 0.060
			$-$ 0.004		$-$ 0.032		$-$ 0.096
	Test	0.939	$+$ 0.003	0.907	0.020	0.404	$+$ 0.053
			$-$ 0.004		$-$ 0.019		$-$ 0.049
Hard negative model	Validation	0.906	$+$ 0.033	*0.943*	$+$ 0.021	0.753	$+$ 0.060
			$-$ 0.040		$-$ 0.030		$-$ 0.096
	Test	0.917	$+$ 0.013	0.926	$+$ 0.011	0.449	$+$ 0.053
			$-$ 0.010		$-$ 0.016		$-$ 0.049
DenseNet-161 with augmentations	Validation	*0.930*	$+$ 0.029	0.922	$+$ 0.018	0.710	$+$ 0.041
			$-$ 0.052		$-$ 0.022		$-$ 0.075
	Test	**0.944**	$+$ 0.014	0.900	$+$ 0.020	0.389	$+$ 0.046
			$-$ 0.008		$-$ 0.017		$-$ 0.041
Ensemble	Validation	0.910	$+$ 0.051	*0.955*	$+$ 0.029	*0.793*	$+$ 0.011
			$-$ 0.037		$-$ 0.038		$-$ 0.009
	Test	0.924	$+$ 0.022	**0.943**	$+$ 0.031	***0.535***	$+$ 0.028
			$-$ 0.039		$-$ 0.031		$-$ 0.025

The highest score for a metric is highlighted in bold for the test set, whereas this is italicised for the validation set. Plus/minus values shows the mean subtracted from the highest and lowest results from the 3-fold experiments, for each model.

### After applying optimal thresholds and post-processing

The post-processing was applied to the results after obtaining optimal thresholds for each fold. In this case, the DenseNet-161 model and the ensemble model. Results are presented in Table 2. From this table it is clear that post-processing has a significant impact, especially on specificity and F1-scores. The best sensitivities for both validation and test sets were found in the DenseNet-161 model results. However, the best performing specificity result was from DenseNet-161 (threshold per fold + post-processed), with 0.992 and 0.984 for validation and test, respectively. As a result, the highest performing test F1-score also came from this model (0.708).

From Table 1, it can be seen that the models on the test data produced sub-optimal F1 scores, suggesting that the models based on a 50% probability decision threshold, did not generalise at an optimal standard. However, Table 2 demonstrates after thresholding and post-processing, the F1 scores significantly improve, suggesting that higher decision thresholds may be required when applied to unseen data. This could be due to textures and different colours presented from the staining process residing in the test data. As a result, these unseen artefacts could lead to an increase in low confidence necrosis predictions, thus producing false positives. This also suggests that structures related to necrosis are learnt well using the training data as true positive necrosis predictions tend to produce high confidence predictions. Table 3 depicts the confusion matrix value results after applying optimal thresholds and the single tile removal post-processing. It can be seen that after applying optimal thresholds and post-processing, the number of false positives (FPs) significantly decrease for both the DenseNet-161 and ensemble models, with a slight reduction in the number of true positives (TPs).

Additionally, accuracy and an average of the sensitivity and specificity (denoted as Sensitivity/Specificity Average) are also introduced into the Table 2. The Sensitivity/Specificity Average can be directly compared to the results from Sharma et al.^22^ where the authors averaged their necrosis and non-necrotic classification results producing 81.44%.When using our dataset, their proposed CNN model produced 83.5% for the Sensitivity/Specificity Average.

Comparatively, our 3-fold scores range from 89.5 to 93.4%, thus producing the highest accuracies for this metric for necrosis versus non necrotic (negative) classification.

**Figure 6:**
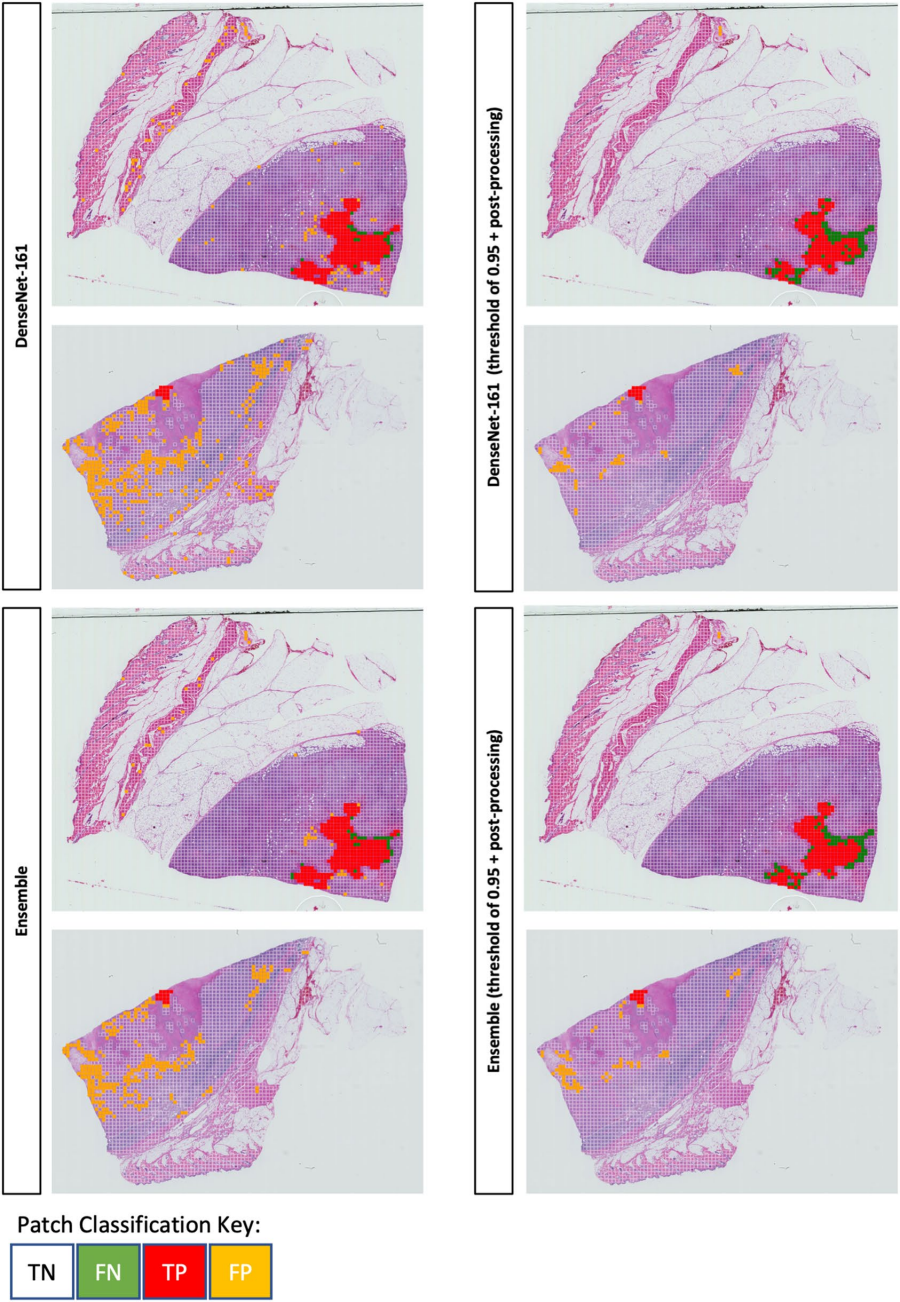
Sample Whole Slide Image (WSI) spatial confusion maps before and after applying optimal threshold (determined from the fold 3 validation set) and post-processing; removing single tile predictions. The left side images shows predictions from the DenseNet-161 and ensemble models with the standard 50% probability decision thresholds. The right side shows predictions after applying the optimal threshold and post-processing. True positives (TP) are displayed in red, false negatives (FN) in green, false positives (FP) in yellow and true negatives (TN) in clear.

A set of exemplar spatial confusion maps are shown in Fig. 5 where we present the results of the tile-based classification overlaid onto the original WSIs created from the hold-out test set are shown in Fig. 6. In this figure, we depict results from the DenseNet-161 model and the ensemble model. The left side shows results with the standard 50% probability threshold applied, whereas the right shows optimal validation threshold applied to the fold 3 test set results, with single tile removal. It is apparent that there are far fewer FPs after the post-processing for both the DenseNet-161 and ensemble models.

The post-processing improved the DenseNet-161 results becoming the top performing model. This is attributed to spatially sparse FP predictions in slides. All models experienced a slight reduction in sensitivity after applying the optimal thresholds and post-processing. There is a slight increase in false negatives, especially around the borders of the necrosis clusters (TPs) in the images. However, the ensemble model spatial confusion matrices depict less FN predictions in the middle of the cluster, in comparison to DenseNet-161. This is important and allows us to understand the limitations of patch-based approaches and may in fact highlight disagreement between annotators. We are aware that boundary cases may exist around the borders of these clusters due to the annotation and patch extraction process. The deep learning models may reflect these uncertainties by producing less confident predictions in these areas. The ensemble model also demonstrates the power of combining multiple different models, mimicking the combination of different ”teachers” or ”experts”, as there are fewer FNs in the middle of the necrosis clusters compared to the DenseNet-161 model.

**Table 2:** 3-fold averaged results after applying optimal thresholds and the single tile removal post-processing to the DenseNet-161, Sharma et al’s^22^ proposed CNN and ensemble models, for the validation and test sets.

Model	Set	Sensitivity		Specificity		F1 score		Accuracy		Sens./ Spec. Avg.	
DenseNet-161	Validation	*0.928*	$+$ 0.029	0.928	$+$ 0.024	0.724	$+$ 0.060	0.927	$+$ 0.017	0.928	$+$ 0.010
			$-$ 0.004		$-$ 0.032		$-$ 0.096		$-$ 0.026		$-$ 0.009
	Test	**0.939**	$+$ 0.003	0.907	$+$ 0.020	0.404	$+$ 0.053	0.908	$+$ 0.019	0.923	$+$ 0.011
			$-$ 0.004		$-$ 0.019		$-$ 0.049		$-$ 0.019		$-$ 0.012
Sharma et al. Proposed CNN	Validation	0.794	$+$ 0.078	0.954	$+$ 0.029	0.727	$+$ 0.031	0.938	$+$ 0.015	0.874	$+$ 0.023
			$-$ 0.097		$-$ 0.031		$-$ 0.020		$-$ 0.021		$-$ 0.034
	Test	0.719	$+$ 0.046	0.951	$+$ 0.004	0.455	$+$ 0.025	0.944	$+$ 0.002	0.835	$+$ 0.023
			$-$ 0.060		$-$ 0.005		$-$ 0.014		$-$ 0.004		$-$ 0.028
DenseNet-161 (threshold per fold + post-processed)	Validation	0.808	$+$ 0.022	*0.992*	$+$ 0.001	0.860	$+$ 0.015	0.973	$+$ 0.003	0.900	$+$ 0.011
			$-$ 0.032		$-$ 0.001		$-$ 0.011		$-$ 0.005		$-$ 0.015
	Test	0.807	$+$ 0.028	**0.984**	$+$ 0.001	**0.708**	$+$ 0.010	**0.978**	$+$ 0.000	0.896	$+$ 0.013
			$-$ 0.032		$-$ 0.001		$-$ 0.011		$-$ 0.000		$-$ 0.016
DenseNet-161 (mean 3-fold threshold + post-processed)	Validation	0.813	$+$ 0.042	0.991	$+$ 0.006	0.855	$+$ 0.020	0.972	$+$ 0.003	0.902	$+$ 0.018
			$-$ 0.069		$-$ 0.005		$-$ 0.016		$-$ 0.005		$-$ 0.032
	Test	0.807	$+$ 0.010	0.983	$+$ 0.004	0.702	$+$ 0.033	0.977	$+$ 0.004	0.895	$+$ 0.005
			$-$ 0.011		$-$ 0.004		$-$ 0.033		$-$ 0.004		$-$ 0.003
Ensemble	Validation	0.910	$+$ 0.051	0.955	$+$ 0.029	0.793	$+$ 0.078	0.950	$+$ 0.022	*0.933*	$+$ 0.006
			$-$ 0.037		$-$ 0.038		$-$ 0.112		$-$ 0.029		$-$ 0.004
	Test	0.924	$+$ 0.022	0.943	$+$ 0.031	0.535	$+$ 0.133	0.943	$+$ 0.029	**0.934**	$+$ 0.011
			$-$ 0.039		$-$ 0.031		$-$ 0.120		$-$ 0.029		$-$ 0.007
Ensemble (threshold per fold + post-processed)	Validation	0.853	$+$ 0.009	0.990	$+$ 0.001	*0.878*	$+$ 0.011	*0.976*	$+$ 0.002	0.922	$+$ 0.004
			$-$ 0.015		$-$ 0.001		$-$ 0.006		$-$ 0.004		$-$ 0.008
	Test	0.846	$+$ 0.050	0.981	$+$ 0.031	0.704	$+$ 0.026	0.977	$+$ 0.003	0.914	$+$ 0.022
			$-$ 0.060		$-$ 0.051		$-$ 0.019		$-$ 0.004		$-$ 0.028
Ensemble (mean 3-fold threshold + post-processed)	Validation	0.868	$+$ 0.070	0.982	$+$ 0.012	0.851	$+$ 0.022	0.969	$+$ 0.006	0.925	$+$ 0.015
			$-$ 0.048		$-$ 0.018		$-$ 0.043		$-$ 0.008		$-$ 0.022
	Test	0.871	$+$ 0.032	0.974	$+$ 0.015	0.673	$+$ 0.089	0.971	$+$ 0.012	0.923	$+$ 0.026
			$-$ 0.059		$-$ 0.014		$-$ 0.085		$-$ 0.012		$-$ 0.018

Presented in this table are the mean sensitivity, specificity and f1-scores averaged across all three folds. ”Threshold per fold + post-processed” is where optimal thresholds derived from three-way cross-validation followed by single tile removal were applied to all validation folds and the hold-out test set. ”Mean threshold + post-processed” is where the optimal thresholds for each fold have been averaged and then applied to each folds validation and hold-out test set. The highest score for a certain metric is highlighted in bold for the test set, whereas it is italic for the validation set. Plus/minus values shows the mean subtracted from the highest and lowest results from the 3-fold experiments, for each model.

**Table 3:** 3-fold averaged confusion matrix value results after applying optimal thresholds and the single tile removal post-processing to the DenseNet-161 and ensemble models, for the hold-out test sets.

Model	TN		TP		FN		FP	
DenseNet-161	30880	$+$ 672	1081	$+$ 3	70	$+$ 5	3171	$+$ 651
		$-$ 651		$-$ 5		$-$ 3		$-$ 672
DenseNet-161 (threshold per fold + post-processed)	33505	$+$ 27	929	$+$ 32	222	$+$ 37	546	$+$ 19
		$-$ 19		$-$ 37		$-$ 32		$-$ 27
DenseNet-161 (mean 3-fold threshold + post-processed)	33479	$+$ 143	929	$+$ 12	222	$+$ 12	572	$+$ 126
		$-$ 126		$-$ 12		$-$ 12		$-$ 143
Ensemble	32119	$+$ 1053	1063	$+$ 26	88	$+$ 44	1932	$+$ 1048
		$-$ 1048		$-$ 44		$-$ 26		$-$ 1053
Ensemble (threshold per fold + post-processed)	33221	$+$ 101	983	$+$ 57	168	$+$ 69	830	$+$ 184
		$-$ 184		$-$ 69		$-$ 57		$-$ 101
Ensemble (mean 3-fold threshold + post-processed)	33180	$+$ 504	1002	$+$ 37	149	$+$ 68	871	$+$ 460
		$-$ 460		$-$ 68		$-$ 37		$-$ 504

Presented in this table are the true negative (TN), true positive (TP), false negative (FN) and false positive (FP) values averaged across all three folds, rounded to the nearest whole figure. Plus/minus values shows the mean subtracted from the highest and lowest results from the 3-fold experiments, for each model.

## Discussion

A necrosis detection method was created after investigating a pretrained DenseNet-161 model, hard negative mining and ensemble models. We further investigated the application of optimal thresholds and further post-processing. This is the first known necrosis detection model for cPWTs and in general cSTS. As a result, we also produce state-of-the-art performance metrics, especially regarding accuracy and sensitivity/ specificity averages for necrosis detection in cPWTs.

The post-processing alongside applying optimal thresholds allowed the DenseNet-161 model to produce the best F1-scores: the key metric for evaluation for this work. This is most likely due to the DenseNet-161 model generating more ”sparse” FP predictions in than the ensemble model, where there are more clustered FP predictions. Nevertheless, although producing the highest F1-scores, this difference was marginally higher than the ensemble model with post-processing.

However, upon inspection of the spatial confusion matrix heatmaps, it was observed that both optimised models differed slightly especially in regards to false negative (FN) predictions, with slightly fewer FNs inside the true positive clusters for the ensemble model. This further demonstrates the difference in learning between alternative types of machine learning models such as deep learning models and ensembles with logistic regression as their backbone. This paper demonstrates that deep learning models can be successfully used as a diagnostic support tool for grading cPWT in cSTS. Necrosis detection should also be investigated with other cSTS subtypes.

The study presented here has the potential to improve the veterinary anatomic pathology workflow. The application of deep learning based-methods to diagnostic veterinary pathology will improve the accuracy of diagnosis and allow pathologist laboratories to handle larger clinical caseloads. These methods could also be applied to other imaging modalities in veterinary health, including clinical veterinary pathology such as cytology and diagnostic imaging such as MRI or radiography.

## Future work

Future work should include exploring further alternative convolutional neural networks that have not been previously and thoroughly investigated for use in digital pathology. Although this necrosis model has been developed and trained using cPWT, it would also be of interest to apply these necrosis detection models to other closely related cSTS subtypes.

## Supplementary Information

Supplementary Information.

## Data Availability

The datasets used and/or analysed during the current study available from the corresponding author on reasonable request.
