# Supplementary material for: Deep learning for necrosis detection using canine perivascular wall tumour whole slide images
Source: Sci Rep. 2022 Jun 23;12:10634. doi: 10.1038/s41598-022-13928-1 (PMC9226022; doi:10.1038/s41598-022-13928-1)
Supplement: Supplementary file 1 — Supplementary Information. [file 41598_2022_13928_MOESM1_ESM.pdf]

Supplementary Information

Table S1 A comparison of the pretrained DenseNet-161 model results with an AlexNet (not pretrained) and the proposed CNN model from Sharma et al.[22]. The table shows sensitivity, specificity, F1-score and Area under the ROC Curve (AUC) results for both validation and test sets.

Model	Set	Sensitivity		Specificity		F1 Score		AUC	
**DenseNet-161**	Validation	*0.928*	+0.029 -0.004	0.928	+0.024 -0.032	0.724	+0.060 -0.096	*97.9*	+0.5 -0.6
	Test	**0.939**	+0.003 -0.004	0.907	+0.020 -0.019	0.404	+0.053 -0.049	**97.6**	+0.5 -0.7
**AlexNet (Not Pretrained)**	Validation	0.873	+0.043 -0.072	*0.955*	+0.033 -0.041	*0.779*	+0.061 -0.070	96.5	+0.5 -0.8
	Test	0.817	+0.020 -0.011	**0.965**	+0.004 -0.003	**0.572**	+0.016 -0.009	96.9	+0.3 -0.4
**Sharma et al.[22] Proposed CNN**	Validation	0.794	+0.078 -0.097	0.954	+0.029 -0.031	0.727	+0.031 -0.020	90.4	+0.9 -1.3
	Test	0.719	+0.046 -0.060	0.951	+0.004 -0.005	0.455	+0.025 -0.014	90.2	+0.6 -0.5

To demonstrate the efficacy of our methods, a comparison between an AlexNet (not pretrained) were implemented and the proposed CNN model by Sharma et al.[22]. For these experiments, the authors suggested using Stochastic Gradient Descent, where we applied their learning rate parameters. We also applied weighted cross-entropy loss to mitigate the class-imbalance to closely reflect the experiments completed by Sharma et al.[22]. Patches were resized to 512 x 512 pixels via bilinear interpolation for Sharma et al's proposed CNN model. Patches were also resized to 227 x 227 pixels as input to the AlexNet model. All models produce relatively low F1-scores at the 50% decision threshold. As stated in the main text, our DenseNet model also provided a higher AUC value prior to thresholding and post-processing. Therefore, the pretrained DenseNet-161 was used as the main choice of model, as it had greater potential for performance improvement via thresholding and post-processing. Additionally, it must be noted that the sensitivities are lower for the alternative models.
